# Macular superficial vascular density on optical coherence tomography angiography in children with unilateral anisometropic and bilateral hyperopic amblyopia

**DOI:** 10.1038/s41598-023-40025-8

**Published:** 2023-08-08

**Authors:** Yeon Woong Chung, Sun Young Shin, Hye Bin Yim

**Affiliations:** 1 Department of Ophthalmology & Visual Science, College of Medicine, St. Vincent’s Hospital, The Catholic University of Korea, Seoul, Republic of Korea; 2 Department of Ophthalmology & Visual Science, College of Medicine, Seoul St. Mary’s Hospital, The Catholic University of Korea, Seoul, Republic of Korea; 3 Department of Ophthalmology & Visual Science, College of Medicine, Incheon St. Mary’s Hospital, The Catholic University of Korea, Seoul, Republic of Korea; 4 Department of Ophthalmology & Visual Science, College of Medicine, Incheon St. Mary’s Hospital, The Catholic University of Korea, #56 Dongsu-ro, Bupyeong-gu, Seoul, 21431 Republic of Korea

**Keywords:** Paediatric research, Visual system

## Abstract

We analyzed whether macular superficial vascular density (SVD) and foveal vascular zone (FAZ) on optical coherence tomography angiography (OCTA) can distinguish between bilateral ametropic and anisometropic amblyopia. We included 42, 33, and 50 eyes in the bilateral ametropic amblyopia, anisometropic amblyopia, and normal control groups, respectively. Using macular swept-source optical coherence tomography angiography, we measured and analyzed the superficial FAZ areas and five sectoral macular SVDs after magnification correction. The anisometropic amblyopic eye group showed significantly increased foveal SVDs (*p* < 0.001) and significantly decreased superficial FAZ areas (*p* < 0.001), compared with the remaining groups. Additionally, the bilateral ametropic amblyopia group had significantly decreased nasal SVDs. SVDs and superficial FAZ areas differed among hyperopic amblyopia subtypes. These findings may reflect vascular distribution differences and macular changes in hyperopic amblyopia subtypes compared with normal eyes.

## Introduction

Amblyopia is a common condition, with a prevalence of 1–4%, that prevents the normal development of visual acuity in one or both eyes because of refractive errors, strabismus, or visual deprivation^1,2^. It has been regarded as a developmental cortical disease of the visual pathway essentially due to abnormal visual stimulus, reaching the binocular cortical cells, which may be multivariate^3,4^. Once cortical changes occur, it entices the visual cortex to prefer one eye over the other, leading to several functional deficiencies in the eye, altered visual function like decreased visual acuity, impaired contrast sensitivity, particularly in detecting high spatial frequency stimuli, and impaired motor signs like hand–eye coordination and spatial localization. It can be either unilateral or bilateral^5^. Amblyopia mainly consists of refractive, strabismic, deprivation, and mixed^6^.

Refractive amblyopia occurs in the presence of large (ametropic) or bilaterally unequal amounts (anisometropic) of refractive errors during childhood. Refractive amblyopia is classified as hyperopic, myopic, astigmatic, or mixed, according to refractive error type; each type has different conditions that may cause amblyopia. Additionally, the causes of ametropic and anisometropic amblyopia may differ even in eyes with the same refractive error type, suggesting that mechanisms other than an optical blur (e.g., abnormal bilateral interactions) are involved^7^.

Optical coherence tomography (OCT) is a powerful noninvasive, high-resolution interferometric imaging technology that allows in vivo cross-sectional visualization of biological tissues^8^; it provides a three-dimensional image of the retina. Several studies involving recent OCT technologies have demonstrated structural abnormalities and modifications in the retina, optic nerve, and choroid in amblyopic eyes, compared with normal controls^9–14^.

Swept-source OCT uses larger wavelengths of infrared light than conventional spectral-domain OCT. These longer wavelengths improve tissue penetration and imaging through optical opacities, are invisible to the patient, provide greater sensitivity for low blood flow, and reduce motion artifacts without compromising the axial resolution^15^. This approach has been applied to OCT angiography (OCTA)^16^, which allows rapid and accurate layer-by-layer visualization of retinal blood vessels and analyses of the retinal microvasculature without requiring the injection of fluorescent dyes^17^. OCTA also offers high intra-visit repeatability and inter-visit reproducibility^18,19^; it has been used for the diagnosis and prognosis of various ophthalmologic diseases^20–26^.

Several studies have compared amblyopic and normal eyes using OCTA^7,27–29^. However, any differences between ametropic and anisometropic amblyopia were not analyzed in previous studies. In eyes with the same type of refractive error, the threshold for ametropic amblyopia and the disparity between the two eyes (anisometropic amblyopia) differ. There is also a need to compare the vascular density observed on OCTA in bilateral ametropic and anisometropic amblyopia with the vascular density in normal eyes. Therefore, the present study investigated whether macular superficial vascular density (SVD) and foveal vascular zone (FAZ) area on OCTA could be used to distinguish between bilateral ametropic and anisometropic amblyopia.

## Methods

### Amblyopic and control group

Children who visited Seoul Saint Mary’s Hospital, South Korea, between March 2018 and December 2019 were recruited for this study. All participants had unremarkable general and ocular health. This study protocol was approved by the institutional review board of the Catholic University of Korea Catholic Medical Center (Seoul Saint Mary’s Hospital), and the study protocol adhered to the tenets of the Declaration of Helsinki. Informed consent was obtained from all subjects and/or their legal guardian(s). All participants underwent a complete ophthalmic examination, including a slit-lamp examination, intraocular pressure assessment, visual acuity measurement, cycloplegic refraction, alternate cover tests, fundus photography, and axial length.

Inclusion criteria were age ≤ 19 years and mean cup-to-disc ratio ≤ 0.5 on fundus photography. Individuals with a refractive error of spherical equivalent ≤  + 3.00 diopters (D), and uncorrected or best-corrected visual acuity (BCVA) ≥ 0.8 in both eyes (measured using a Snellen chart), were included in the normal control group. The bilateral amblyopia group was defined as newly diagnosed children with refractive error of hyperopia ≥ 4.00 D, astigmatism ≤ 2.00 D, BCVA < 0.8, and a BCVA difference of < two lines between the eyes. The anisometropic amblyopia group included newly diagnosed children with an interocular hyperopic difference of refractive error ≥ 1.50 D, BCVA difference ≥ two lines, and BCVA ≥ 0.8 in the normal eye.

Exclusion criteria were intraocular pressure > 21 mmHg, assessed using pneumatic tonometry (CT-80 non-contact computerized tonometer; Topcon Corp., Tokyo, Japan), as well as strabismus, developmental delays, neurological impairment, previous surgery, and other visual pathway or ocular diseases.

**Figure 1:**
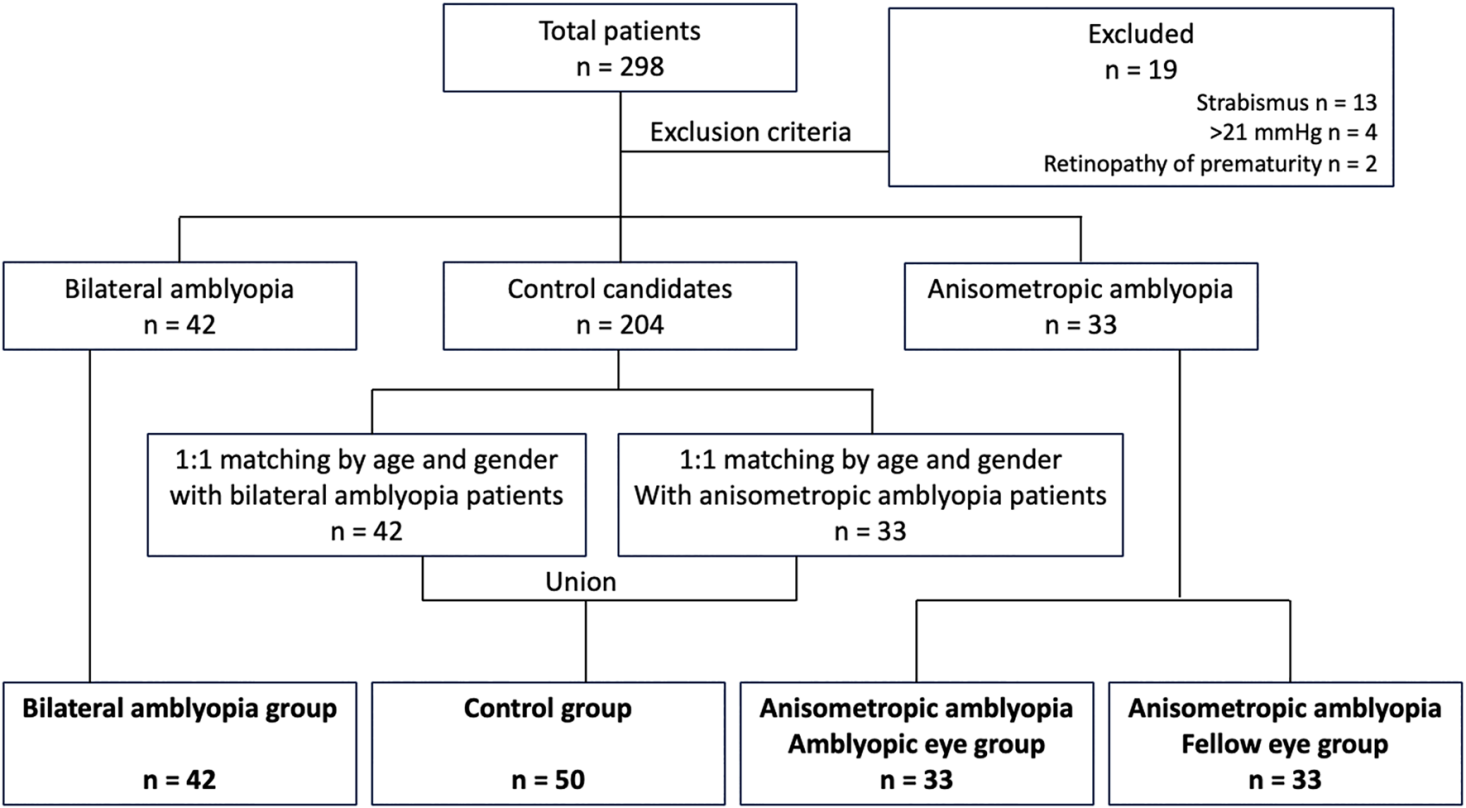
The sequential flow for group selection.

Participants in the normal control group were matched for age and compared with the bilateral and anisometropic amblyopia groups using MatchIt library in R software, version 3.6 (The R Foundation, Vienna, Austria). Of the 204 control candidates, we selected a control group that matched 1:1 with the bilateral amblyopia group and another one that matched 1:1 with the anisometropic amblyopia group. Then, the two control groups were combined in a union as the normal control group (Fig. 1).

### Data acquisition

Experienced examiners obtained photographs of the optic disc using a fundus camera (Nonmyd 7; Kowa, Tokyo, Japan) and a digital camera (D70s; Nikon, Tokyo, Japan). Images were captured using a program provided by Kowa. A single specialist (SYS) determined the cup-to-disc ratios. Axial lengths (ALs) were measured by a single observer, using an IOLMaster (Carl Zeiss AG, Oberkochen, Germany).

**Figure 2:**
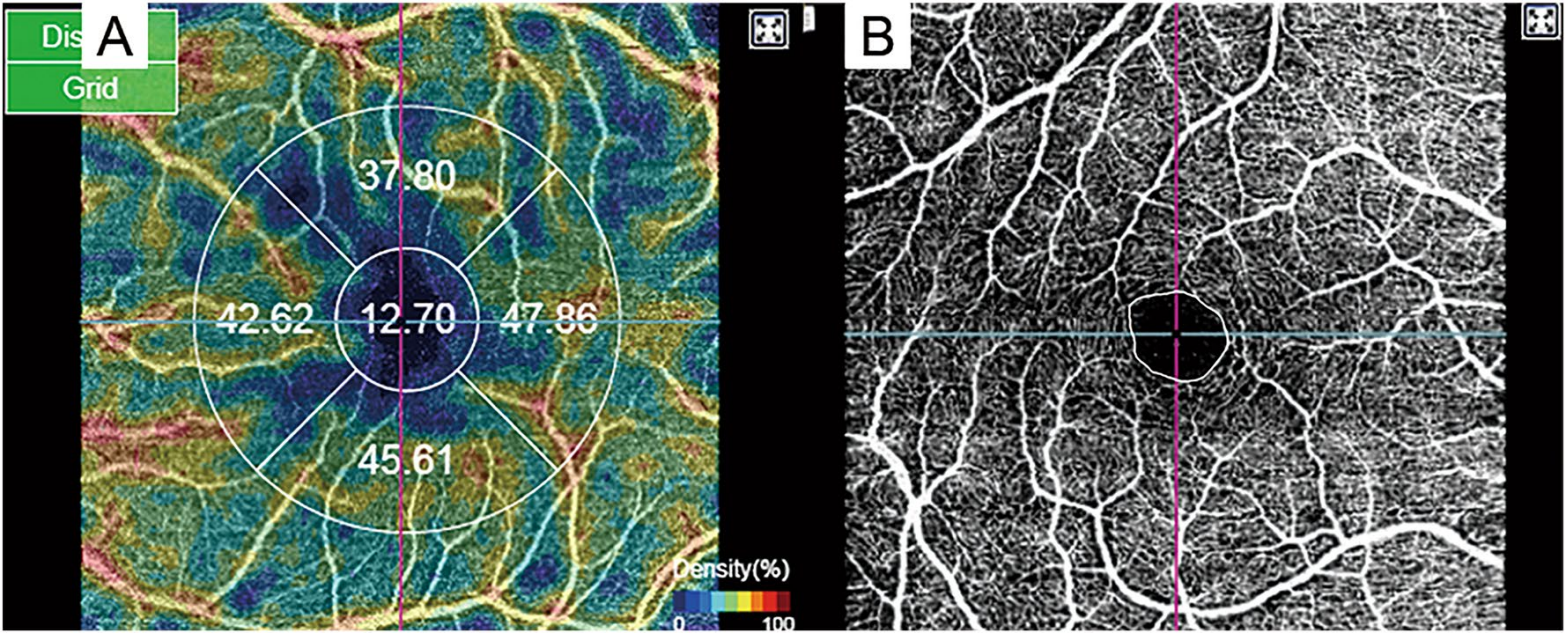
Representative image of (**A**) superficial vascular density and (**B**) superficial foveal avascular zone area in the ocular coherence tomography angiography.

All these patients subsequently underwent swept-source OCTA scans of the macula. (4.5 × 4.5 mm scan; Topcon DRI Triton Swept-source OCT; Topcon, Tokyo, Japan); a single observer performed these examinations. Based on a 3 × 3 mm macular scan, vascular density was measured along the superficial retinal plexus using a built-in proprietary software. Only patient data whose OCTA image quality was more than 40 was collected as mentioned previously^30^. Density values along the superior, inferior, nasal, and temporal quadrants, as well as the foveal area defined as the central 1-mm circle on the macular scan were measured (Fig. 2A). The deep plexus density was not compared because it could not be quantified using current-generation OCTA. FAZ areas were automatically calculated by the internal logic after its boundaries had been determined using a built-in tool (Fig. 2B). Then, FAZ areas were re-calculated after correcting the sizes through magnification correction using Littmann’s formula modified by Bennett as follows^31,32^.

$$Modified \; diameter=p \times 0.01306 \times \left(AL-1.82\right) \times scan \;diameter$$

where *p* is the magnification factor, which was regarded as 3.3820 based on a previous analysis using the same device^33^.

### Statistical analysis

Quantitative variables were expressed as means ± standard errors. Data were compared by analysis of variance after a normal distribution had been confirmed using the Shapiro–Wilk test. Post hoc analysis was performed with Tukey’s honestly significant difference test. Statistical analyses were performed using R software, version 3.6 (The R Foundation). The threshold for statistical significance was set at *p* < 0.05.

## Results

In total, 125 eyes in 125 children (44 males and 81 females) were included; 42 in the bilateral amblyopia group, 33 in the anisometropic amblyopia group, and 50 in the normal control group. The descriptive statistics are shown in Table 1. There were no statistically significant differences in age and sex among the groups.

**Table 1:** Optical coherence tomography angiography parameters.

	Bilateral amblyopia	Anisometropic amblyopia		Control	*p*-value
		Amblyopic	Fellow		
Number	42	33		50	
Age (y)	6.33 ± 2.71	7.71 ± 3.38		6.76 ± 2.27	0.254*
Sex (M/F)	16/26	12/21		16/34	0.672^†^
Corrected visual acuity (Snellen)	0.48 ± 0.21	0.41 ± 0.35	0.87 ± 0.04	0.91 ± 0.06	< 0.001*
Refractive error (SE)	+ 4.38 ± 2.42	+ 3.86 ± 3.11	+ 1.55 ± 1.23	+ 1.45 ± 2.10	0.012*
Axial length (mm)	20.98 ± 1.81	21.22 ± 1.16	22.04 ± 1.21	22.08 ± 1.70	< 0.001*

SE: spherical equivalent.

*Analysis of variance, ^†^Chi-square test.

**Figure 3:**
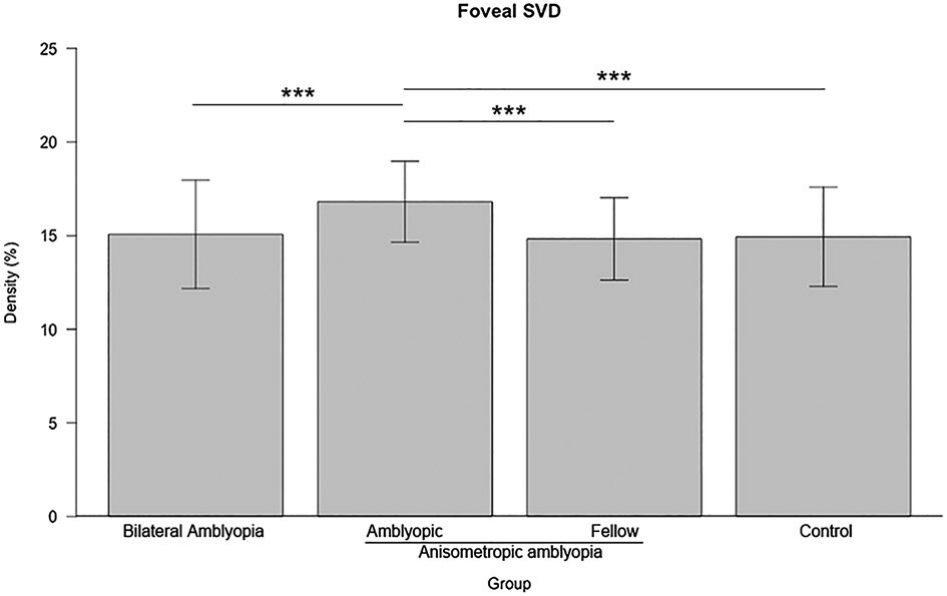
Comparison of the foveal superficial vascular density among groups. ***P < 0.001 on post hoc analysis.

**Figure 4:**
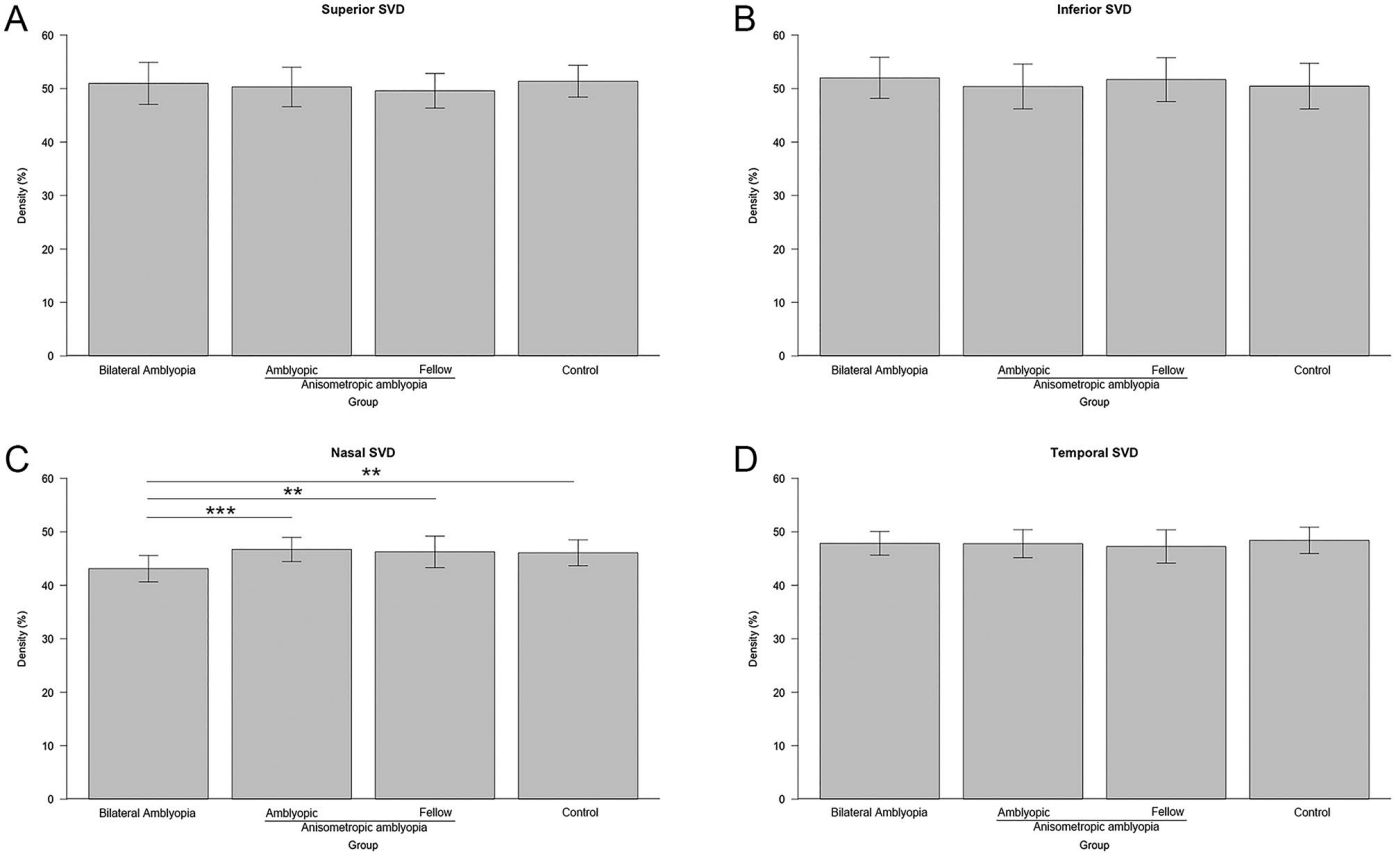
Comparison of parafoveal superficial vascular density (SVD) among groups. (**A**) superior, (**B**) inferior, (**C**) nasal, and (**D**) temporal SVDs. Only the bilateral amblyopia group had significantly decreased nasal superficial vascular density compared to the normal control group (*p* = 0.006), the amblyopic eye (*p* < 0.001), and the fellow eye group (*p* = 0.002) in the anisometropic amblyopia. **P < 0.01 and ***P < 0.001 when the post hoc analysis compared the two groups.

Table 2 shows the foveal and parafoveal sectoral SVDs. Foveal and nasal SVDs significantly differed among the groups (*p* = 0.027 and *p* = 0.003). Post hoc analysis revealed that the anisometropic amblyopia group had significantly increased foveal SVD, compared with the remaining groups (*p* < 0.001; Fig. 3). Additionally, the bilateral amblyopia group had significantly decreased nasal SVD (Fig. 4C), compared with the remaining groups. On the other hand, the other sectoral SVDs did not show significant statistical differences among the groups (Fig. 4A,B,D).

**Table 2:** Superficial vascular density values among groups.

Superficial vascular density	Bilateral amblyopia	Anisometropic amblyopia		Control	*p*-value*
		Amblyopic	Fellow		
Foveal	15.07 ± 2.89	16.81 ± 2.16	14.83 ± 2.20	14.94 ± 2.65	0.027
Superior	50.97 ± 3.94	50.30 ± 3.70	49.59 ± 3.24	51.37 ± 2.97	0.28
Inferior	52.01 ± 3.84	50.39 ± 4.18	51.68 ± 4.11	50.45 ± 4.26	0.26
Temporal	47.84 ± 2.21	47.77 ± 2.62	47.26 ± 3.11	48.41 ± 2.46	0.18
Nasal	43.11 ± 2.46	46.71 ± 2.26	46.26 ± 2.95	46.08 ± 2.42	0.003

*Analysis of variance.

**Figure 5:**
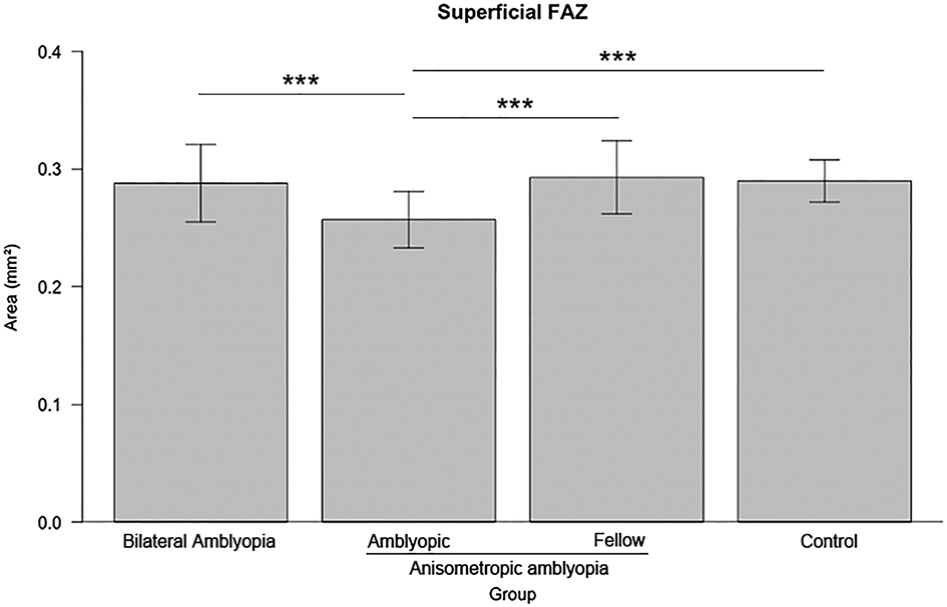
Comparison of the superficial foveal avascular zone area among groups. The area was significantly decreased in the anisometropic amblyopia group, compared with other groups. ***P < 0.001 when the post hoc analysis compared the two groups.

The anisometropic amblyopia group had significantly decreased superficial FAZ area, compared with the other groups (Table 3 and Fig. 5). All groups had moderately negative correlations with foveal SVD (Table 3).

**Table 3:** Comparison of foveal avascular zone areas among groups and correlation with foveal superficial vascular density.

	Bilateral amblyopia	Anisometropic amblyopia		Control	*p*-value*
		Amblyopic	Fellow		
Area					
Superficial FAZ (mm^2^)	0.288 ± 0.033	0.257 ± 0.024	0.293 ± 0.031	0.290 ± 0.018	0.016
Correlation with superficial foveal SVD	− 0.54 ± 0.08	− 0.52 ± 0.10	− 0.53 ± 0.13	− 0.55 ± 0.09	
* p*-value^†^	< 0.001	< 0.001	< 0.001	< 0.001	

FAZ: foveal avascular zone; SVD: superficial vascular density.

*Analysis of variance, ^†^Pearson’s correlation test.

## Discussion

To our knowledge, the present study represents the first analysis of differences in SVD according to hyperopic refractive amblyopia subtype, compared with normal eyes. Anisometropic amblyopic eyes showed increased foveal SVDs, compared with normal and healthy fellow eyes. They also had significantly decreased superficial FAZ areas, compared with the other groups.

A previous study demonstrated that the superficial FAZ area was significantly smaller in amblyopic eyes than in fellow eyes. There was no significant difference in the total macular SVD between amblyopic and fellow eyes after magnification correction^34^. In contrast, the present study measured foveal and four parafoveal sectoral SVDs. Except for foveal SVD, the SVDs showed no significant differences. Thus, the previous results were partially consistent with our findings, but the present study provided more detailed results.

Previous studies reported that the FAZ area in anisometropic amblyopic eyes did not significantly differ from the FAZ area in fellow or normal eyes^27,28,35,36^. However, there remains controversy regarding the relationship between amblyopic eyes and SVD, as measured using OCTA. Some studies have shown significantly lower SVDs in amblyopic patients, compared with normal individuals^27,35,37,38^. However, those studies did not perform magnification correction. Sampson et al. demonstrated the effects of AL variation-related image magnification on SVD and FAZ area measurements in OCTA examinations^39^. They reported that image size correction in foveal SVD and FAZ area measurements was > 5% in 51% and 74% of the eyes, respectively. In contrast, there was < 5% correction in parafoveal SVD measurements, indicating that FAZ area and foveal SVD may be overestimated in eyes with shorter ALs (e.g., hyperopic eyes). In the present study, only hyperopic amblyopic eyes were included. Additionally, bilateral amblyopic eyes did not show any differences in foveal SVDs and FAZ areas, compared with fellow eyes in anisometropic amblyopia and normal eyes, despite shorter ALs and correction for magnification error. Therefore, the results of this study, obtained via magnification correction, are more suitable for drawing appropriate conclusions.

The bilateral amblyopic eye group showed decreased nasal SVDs, compared with the control group. Based on an analysis of bilaterally amblyopic children, Lonngi et al. concluded that SVDs on the 6 × 6-mm scans were significantly different between amblyopic and normal eyes^27^. However, their study included both anisometropic and bilateral amblyopic children. They also did not localize the SVD area and used total SVD. Because anisometropic amblyopic eyes did not show a significant difference in nasal SVD compared with fellow and normal eyes in the present study, bilateral amblyopic eyes may require greater blood supply from the nasal side of macula for normal visual development. If these findings are confirmed hemodynamically in additional experiments or large-scale studies, clinicians may predict the treatment effects of children with hyperopic amblyopia who are poor at expressing their visual acuity in the clinic by using perfusion changes at the particular macular section as an objective prognostic factor and reduce the amount of time to continue unnecessary amblyopia treatments such as occlusion patch.

The present study had some limitations. First, the number of participants in each group was relatively small. Analyses of larger samples may lead to different conclusions. However, there were no statistically significant differences in age and sex among the groups, and bias was reduced by correction for image magnification errors. Second, macular thickness using OCT was not assessed because a lack of cooperation led to OCT performance in only a small percentage of the children. Further analyses, including assessments of macular thickness, may have yielded even richer results.

In conclusion, SVDs and FAZ areas differed among hyperopic amblyopia subtypes. Anisometropic amblyopic eyes had larger foveal SVDs and smaller FAZ areas, compared with fellow and normal eyes. Bilateral amblyopic eyes had greater nasal SVDs than normal eyes. These findings suggest hemodynamic distribution and macular changes among hyperopic amblyopia subtypes.

## Data Availability

The datasets used and/or analyzed during the current study available from the corresponding author on reasonable request.
